# Modelling extracellular matrix and cellular contributions to whole muscle mechanics

**DOI:** 10.1371/journal.pone.0249601

**Published:** 2021-04-02

**Authors:** Ryan N. Konno, Nilima Nigam, James M. Wakeling

**Affiliations:** 1 Department of Mathematics, Simon Fraser University, Burnaby, British Columbia, Canada; 2 Department of Biomedical Physiology and Kinesiology, Simon Fraser University, Burnaby, British Columbia, Canada; University of Zaragoza, SPAIN

## Abstract

Skeletal muscle tissue has a highly complex and heterogeneous structure comprising several physical length scales. In the simplest model of muscle tissue, it can be represented as a one dimensional nonlinear spring in the direction of muscle fibres. However, at the finest level, muscle tissue includes a complex network of collagen fibres, actin and myosin proteins, and other cellular materials. This study shall derive an intermediate physical model which encapsulates the major contributions of the muscle components to the elastic response apart from activation-related along-fibre responses. The micro-mechanical factors in skeletal muscle tissue (eg. connective tissue, fluid, and fibres) can be homogenized into one material aggregate that will capture the behaviour of the combination of material components. In order to do this, the corresponding volume fractions for each type of material need to be determined by comparing the stress-strain relationship for a volume containing each material. This results in a model that accounts for the micro-mechanical features found in muscle and can therefore be used to analyze effects of neuro-muscular diseases such as cerebral palsy or muscular dystrophies. The purpose of this study is to construct a model of muscle tissue that, through choosing the correct material parameters based on experimental data, will accurately capture the mechanical behaviour of whole muscle. This model is then used to look at the impacts of the bulk modulus and material parameters on muscle deformation and strain energy-density distributions.

## Introduction

Skeletal muscle is a complex heterogeneous structure, and a three dimensional continuum model is required to capture its complete mechanics. One dimensional models have been developed, often to describe whole body movement or inter-muscular dynamics (eg. [1]). However, when examining the mechanics or force production of the muscle these models are not sufficient to understand the complex effects from the heterogeneity or architecture of muscle [2]. In fact, three dimensions are required to fully capture the bulging and deformation seen in skeletal muscle [3]. Therefore, to capture the complex aspects of muscle tissue, these models are typically built using the theory of continuum mechanics and solved using a finite element method [4–9].

Muscle is composed of many components making it a highly heterogeneous structure, and these aspects are typically investigated in micro-mechanical [10–14] and homogenization [15–17] models. The tissue heterogeneity effects are often related to micro-structure [18, 19], and these effects cannot be implemented using a single-scale model. The micro-mechanical components of muscle are those that are visible on a microscopic level and contribute to the mechanical behaviour of muscle tissue. There are many micro-mechanical components of muscle aside from the contractile fibres alone. In particular, muscle consists of connective tissue, fluid, cellular components, and muscle fibres which make it a highly heterogeneous material. Skeletal muscle consists of muscle fibres surrounded by a layer of connective tissue (endomysium), and groups of fibres are bundled together into muscle fascicles by another layer of connective tissue (perimysium). Bundles of fascicles are what composes the muscle volume and is held together with a third layer of connective tissue (epimysium) [18, 19]. The combination of connective tissue layers forms the extra-cellular matrix (ECM) and is typically less than 10% of the muscle volume in healthy muscle [20], however the ECM has been shown to have a large impact on the muscle force development [21]. The reason for this is the high degree of structure found in the ECM along with the stiff collagen fibres, which results in a significant contribution to the passive stiffness of the muscle [19, 22–27].

In order to capture the complex effects of the micro-mechanical factors on a whole muscle level, a principled approach needs to be taken. This procedure will allow for consideration of microscopic properties and their effects on the macroscopic muscle model. The micro-mechanical influences on whole muscle effects have been investigated in many studies (eg. [10–12, 14, 15, 17]). A study by Ceelen et al. [12] developed a micro-mechanical model of skeletal muscle for an analysis of the effect of deformation induced hypoxic damage. Sharafi and Blemker [10] developed a formulation of the micro-mechanical effects for healthy muscle that could be implemented in a macroscopic model. Work by Rhörle et al. [13] produced a multi-scale framework for a continuum mechanical model, and included the effects from motor unit recruitment and allows for analysis of electro-physiological behaviour. These developments however do not allow for simple application to the macroscopic level, and hence studies combining the material effects into a homogenous macroscopic model have been performed [15–17]. By performing these homogenizations, a better understanding of the mechanical properties can be obtained in microscopically altered muscle tissue, such as fibrotic tissue that can result from muscular dystrophies, cerebral palsy, and aging [22, 28].

In this study, a principled approach will be taken to develop an isotropic aggregate material that will give a representation of the micro-mechanical effects that can be modelled on a macroscopic level. This homogenization will take into account two factors: a cellular component including the fibres and other cellular materials, and an ECM component. The cellular materials being considered are both effects from cells external to the muscle fibres (eg. satillite cells, nerve bodies, capilleries), as well as the intracellular effect from the fibres aside from the myofibrils. Parameters can be developed independently, so that volume changing as well as isochoric properties can be modified. Additionally, this model will differ from previous homogenization studies (eg. [15, 17]) by considering a nonlinear Yeoh model [29] for the cellular component. Due to the lack of cellular component data, mechanical properties from the cells in brain grey matter will be chosen given the material is composed of the neuron cell bodies. This gives grey matter a nonlinear isotropic response [30, 31], and since this is a collection of cells and similar to the the model’s cellular component, these data will be considered. Any anisotropy typically seen in skeletal muscle will be captured in the one dimensional along-fibre component that takes into account the effects from myofilaments within the muscle fibres, and anisotropy conferred by the ECM. Recent experiments have reported varying muscle volume levels over long contractions [32], and changes in volume have been shown to impact passive muscle tension [33]. The distribution of strain energy-densities has been shown to allow for a deeper understanding of the underlying physics of skeletal muscle mechanics [34]. Therefore, the purpose of this study is to develop a principled model that can be accurately fit to existing experimental data, and can then be used to develop a greater understanding of muscle mechanics in response to altered micro-mechanical properties. In particular, we will look at the impact of the stiffness, volume fraction, and bulk moduli of the individual components in the model through a comparison to experimental data and strain energy-density distribution analysis.

## Model

### Continuum mechanical formulation

Continuum mechanics is an effective method to model the physics of biological materials, and is typically used in three dimensional skeletal muscle models [4–6, 13, 15, 17]. To characterize the deformation of a body, Ω_0_, to a new deformed state, Ω, we can introduce the deformation gradient, **F**. The deformation gradient can be defined as

$$\begin{array}{c} \text{F}=\frac{dx}{d\text{X}} \end{array}$$ (1)

where *d* **X** is a line element in the original reference configuration and *d* **x** is a line element in the deformed current configuration. **F** contains the information about how the original configuration is deformed, via rotations or stretches, to get to the current configuration. The dilatation of the material can be denoted as *J* = det(**F**), and remains close to 1.

To characterize the response of a material to deformation, the constitutive laws for the material need to be determined. To do this, stress and strain tensors need to be defined. The model developed here will consider the left Cauchy-Green strain tensor **b** = **FF**^*T*^ to characterize the strain in the material. Skeletal muscle can be modelled using a nonlinear hyperelastic approach. For a hyperelastic material, the formulation of the constitutive relationships can be performed in terms of a strain-energy function which can be calculated at each material point, **X**. The strain-energy function can be represented in the reference configuration as *W*(**X**, **b**) ≡ *W*(**b**). Characterizing the material in terms of the strain-energy allows us to write the constitutive law in terms of the Cauchy Stress Tensor, *σ*, and the left Cauchy-Green strain tensor,

$$\begin{array}{c} \sigma \left(\text{b}\right)=2J^{-1}\text{b}\frac{\partial W(\text{b})}{\partial \text{b}}. \end{array}$$ (2)

In order to determine the constitutive law explicitly, the exact form of *W*(**b**) needs to be determined. For skeletal muscle the strain-energy function can be broken into a volumetric and isochoric component.

$$\begin{array}{c} W_{\text{muscle}}=W_{\text{vol}}\left(J\right)+W_{\text{iso}}\left(\bar{\text{b}}\right) \end{array}$$ (3)

where $\bar{\text{b}}$ is the isochoric component of the left Cauchy-Green Strain tensor, and is defined as $\bar{\text{b}}=J^{-2/3}\text{b}$. The strain-energy function for skeletal muscle, *W*_muscle_(**b**), is composed of the three dimensional base material component, *W*_BM_(**b**), and an along-fibre component, *W*_fibre_(λ), which depends on the local fibre stretch (λ = ||**Fa**_0_||) along the direction of the muscle fibres. **a**_0_ denotes the initial fibre direction with unit length at a given point and ||(⋅)|| denotes the usual *L*^2^ norm of (⋅). Therefore, the volumetric and isochoric components are

$$\begin{array}{c} \begin{array}{cc} W_{\text{iso}} & =W_{\text{fibre}}\left(\lambda \right)+W_{\text{BM},\text{iso}}\left(\bar{\text{b}}\right),\\ W_{\text{vol}} & =W_{\text{BM},\text{vol}}\left(J\right). \end{array} \end{array}$$ (4)

The continuum mechanical formulation developed here can be implemented into a three dimensional finite element model using a three field formulation with the unknowns being the displacement **u**, pressure *p*, and dilation *J* [35]. The Principle of Stationary Energy can be applied to the problem by taking the first variation of the total energy. This gives a nonlinear problem that can be solved using the finite element library Deal ii [36]. Details on the implementation and finite element method can be found in Domínguez [37] and S1 Appendix.

### A principled approach to muscle base material

#### Formulation of the base material

Muscle is often modelled as a fibre reinforced material [4, 6, 7], and so the model developed in this study will consider the muscle as a three dimensional isotropic material with one dimensional fibres running along the length of the muscle belly. The one dimensional along-fibre component is designed to account for any anisotropic effects in the direction of the muscle fibres. In particular, this includes the passive along-fibre effects from within the sarcomeres, such as from the protein titin, and active forces developed between actin and myosins. To analyze the micro-mechanical properties in whole muscle, the isotropic base material can then be constructed by combining the effects from the principle components (ECM and cellular materials). Since the base material has both isochoric and volumetric parts, the a homogenization will need to occur in both of these strain-energy components.

The base material can be formulated by considering a representative volume element (RVE) that encompasses a region, *V*_RVE_, in the initial reference configuration. Since the RVE consists of each of the micro-mechanical components of the muscle, a portion of it will consist of ECM, *V*_ECM_. The rest of the volume will consist of the cellular component, *V*_cell_. Let |*V*_RVE_| denote the volume the region *V*_RVE_, then the volume fraction of each material can be defined as

$$\begin{array}{c} \frac{|V_{\text{ECM}}|}{|V_{\text{RVE}}|}=\alpha ,\;\frac{|V_{\text{cell}}|}{|V_{\text{RVE}}|}=1-\alpha . \end{array}$$ (5)

The volume fractions, *α* and 1 − *α*, are determined in the reference configuration of the RVE, and we assume these volume fractions do not change as the muscle deforms. Since skeletal muscle achieves near incompressibility, this is an accurate approximation to leading order.

The total energy of the RVE, *E*_RVE_, can be written in terms of the microscopic strain-energy functions for the ECM and cellular components as

$$\begin{array}{c} \begin{array}{cc} E_{\text{RVE}}\left(\text{b}\right) & =\int_{V_{\text{RVE}}}W_{\text{RVE}}\left(\text{b}\right)\;dV\\ & =\int_{V_{\text{ECM}}}s_{\text{ECM}}W_{\text{ECM},\text{RVE}}\left(\text{b}\right)\;dV+\int_{V_{\text{CELL}}}W_{\text{CELL},\text{RVE}}\left(\text{b}\right)\;dV \end{array} \end{array}$$ (6)

where *s*_ECM_ is a structural area parameter that is constant over the RVE and will be discussed in more detail in the next section. Dividing by |*V*_RVE_| gives

$$\begin{array}{c} \begin{array}{cc} \frac{1}{|V_{\text{RVE}}|}\int_{V_{\text{RVE}}}W_{\text{RVE}}\left(\text{b}\right)\;dV & =\frac{1}{|V_{\text{RVE}}|}\int_{V_{\text{ECM}}}s_{\text{ECM}}W_{\text{ECM},\text{RVE}}\left(\text{b}\right)\;dV\\ & \;+\frac{1}{|V_{\text{RVE}}|}\int_{V_{\text{CELL}}}W_{\text{CELL},\text{RVE}}\left(\text{b}\right)\;dV\\ & =\alpha \frac{1}{|V_{\text{ECM}}|}\int_{V_{\text{ECM}}}s_{\text{ECM}}W_{\text{ECM},\text{RVE}}\left(\text{b}\right)\;dV\\ & \;+\left(1-\alpha \right)\frac{1}{|V_{\text{CELL}}|}\int_{V_{\text{CELL}}}W_{\text{CELL},\text{RVE}}\left(\text{b}\right)\;dV. \end{array} \end{array}$$ (7)

Given that the RVE is microscopic in size, the following approximations were made

$$\begin{array}{c} \begin{array}{cc} W(\text{b}) & \approx \frac{1}{|V_{\text{RVE}}|}\int_{V_{\text{RVE}}}W_{\text{RVE}}\left(\text{b}\right)\;dV\\ W_{\text{ECM}}\left(\text{b}\right) & \approx \frac{1}{|V_{\text{ECM}}|}\int_{V_{\text{ECM}}}W_{\text{ECM},\text{RVE}}\left(\text{b}\right)\;dV\\ W_{\text{CELL}}\left(\text{b}\right) & \approx \frac{1}{|V_{\text{CELL}}|}\int_{V_{\text{CELL}}}W_{\text{CELL},\text{RVE}}\left(\text{b}\right)\;dV \end{array} \end{array}$$ (8)

where **b** denotes the left Cauchy-Green strain tensor at the centroid of the RVE. This is the familiar Voigt approximation used in skeletal muscle homogenization studies [15, 16].

It then follows that the strain-energy function for the macroscopic base material can be written as a linear combination of the strain energies from each component:

$$\begin{array}{c} W_{\text{BM},\text{iso}}\left(\text{b}\right)=\alpha s_{\text{ECM}}W_{\text{ECM}}\left(\text{b}\right)+\left(1-\alpha \right)W_{\text{cell}}\left(\text{b}\right). \end{array}$$ (9)

Similarly, it is possible to decompose the volumetric strain-energy function into its micro-mechanical components. The volumetric strain-energy component will be characterized using a strain-energy function typically used for soft biological materials [38], and the aggregate function is given as

$$\begin{array}{c} W_{\text{BM},\text{vol}}\left(J\right)=\frac{1}{4}\left(J^{2}-1-2\;\text{log}\left(J\right)\right)\left[\alpha \kappa_{\text{ECM}}+\left(1-\alpha \right)\kappa_{\text{cell}}\right]. \end{array}$$ (10)

The bulk moduli, *κ*_ECM_ and *κ*_cell_, are parameters that will impact the compressibility of the model and can be varied independently for each component.

#### Micro-mechanical components

The strain-energy function for the ECM, *W*_ECM_(**b**), can be determined using experimental data from Gillies et al. [39], which obtained stress-strain curves for decellularized skeletal muscle tissue. These data are used as they are the only mechanical data available for the entire ECM. Other micro-mechanical models consider the response from the isotropic component of the ECM to be of the same order of magnitude [15] as that of the fibres, instead, in this model we consider experimental data for the ECM that have been measured for a decellularized matrix. Additional data for the ECM component will allow for a more accurate representation in the model. Due to difficulty in measuring the decellularized cross-sectional area of the ECM, the stress-strain relationship is typically reported with respect to the cross-sectional area of the muscle tissue. Therefore, to account for this cross-sectional area calculation in the model, the additional coefficient, *s*_ECM_, is used.

Material data for the cellular (non-contractile and non-ECM) properties of skeletal muscle are not available. Some studies have been able to measure properties of isolated muscle fibres. However, tensile data are only available for the longitudinal direction of the muscle fibres [40], which may not necessarily represent the response in the transverse direction. Ideally, data for the cellular component of the base material would be tensile data for fibre and other cellular components measured transverse to the fibre orientation. Since these data are not available other data need to be considered, such as the mechanical behavior of brain grey matter or liver tissue. These materials are considered since they are essentially a cellular mass with no collagen fibres and are often modelled as non-linear isotropic materials [30, 31, 41–43]. Along fibre data have been considered in the homogenization models by Bleiler et al. [17] and Spyrou et al. [15], however, they only considered a linear stress-strain response shown by Smith et al. [28]. Since the liver material has been shown to have some anisotropy, which is likely due to micro-structural effects, grey matter is used for the cellular component.

In order to implement the experimental data for the ECM and cellular components into the model a strain-energy function needs to be used for each of the components. The Yeoh model (Eq 11) gives the energy associated with a deformation in terms of the first invariant of $\bar{\text{b}}$ [29].

$$\begin{array}{c} W\left(\bar{\text{b}}\right)=c_{1}\left(I_{1}-3\right)+c_{2}{\left(I_{1}-3\right)}^{2}+c_{3}{\left(I_{1}-3\right)}^{3} \end{array}$$ (11)

**Fig 1:**
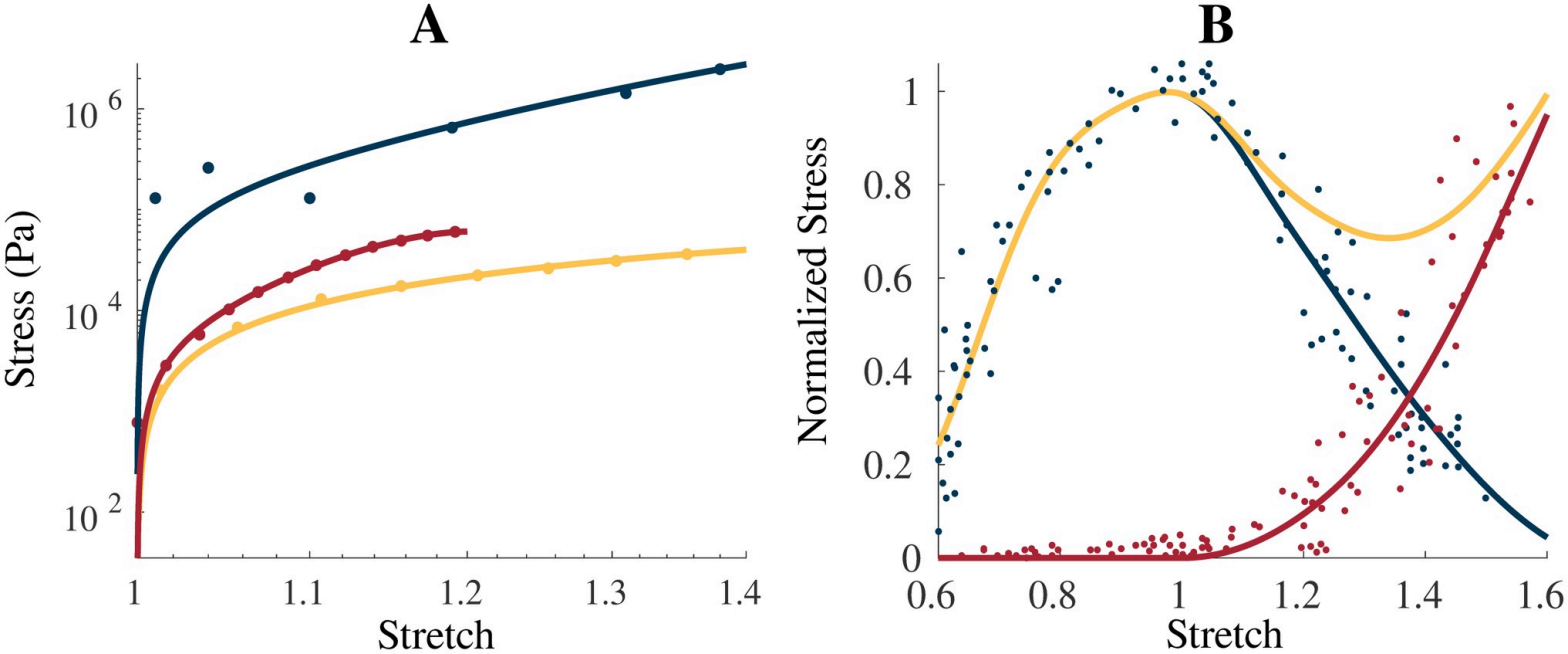
Intrinsic model properties. **(A)** shows the uniaxial stress-stretch relationship for the intrinsic properties of the homogenization: ECM (blue), cellular (yellow), and averaged whole muscle components (red), along with experimental data from Gillies et al. [39] (ECM, blue dot), Jin et al. [30] (brain grey matter, yellow dot), and Mohammadkhah et al. [44] (transverse muscle response, red dot). The averaged whole muscle component was fit to experimental data and is shown for comparison. Total (yellow), passive (red), and active (blue) stress-stretch relationships are shown for the along-fibre response in **(B)** with the experimental data obtained by Winters et al. [45] and normalized to *σ*_0_ = 2 × 10^5^ Pa. ECM component was scaled by 200 in (A) to account for cross-sectional area calculations.

This provides a computationally simplistic model that can sufficiently capture the isotropic behaviour of these components as demonstrated by *r*^2^ values of 0.998 and 0.999 for the ECM and cellular material, respectively (Fig 1A). Fig 1 illustrates the fit of the model for the intrinsic micro-mechanical properties (ECM and cellular components) used in the model along with the experimental whole muscle data from Mohommadkhah et al. [44]. *s*_ECM_ was set at 200 in Fig 1 to account for the aforementioned cross-sectional area calculation effects. This gives a significantly stiffer curve for the ECM compared to both the whole muscle data and the cellular component (Fig 1(A)), which is expected.

### Implementation of the along-fibre component

The along-fibre component of the model was obtained through fitting to experimental data by Winters et al. [45] and is shown in Fig 1(B) in terms of its stress-stretch relationship. The stress response for the passive component of the fibres is given as

$$\begin{array}{c} \sigma_{\text{pass}}\left(\lambda \right)=\sigma_{0}\{\begin{array}{cccc} & 0 & & 0\leq \lambda \leq 1.0\\ & 2.353{\left(\lambda -1.0\right)}^{2} & & 1.0\leq \lambda \leq 1.25\\ & 3.44{\left(\lambda -1.25\right)}^{2}+1.18\left(\lambda -1.25\right)+0.147 & & 1.25\leq \lambda \leq 1.5\\ & 0.427{\left(\lambda -1.5\right)}^{2}+2.90\left(\lambda -1.5\right)+0.656 & & 1.5\leq \lambda \leq 1.65\\ & 3.023(\lambda -1.65)+1.1 & & \lambda >1.65, \end{array} \end{array}$$ (12)

and the active component of the fibres as

$$\begin{array}{c} \sigma_{\text{act}}\left(\lambda \right)=\{\begin{array}{cccc} \sigma_{0} & \left(0.642\;\text{sin}(1.29\lambda +0.629\right)\\ & +0.325\;\text{sin}(5.31\lambda -4.52)\\ & +0.328\;\text{sin}(6.74\lambda +1.69)\\ & +0.015\;\text{sin}(19.8\lambda -7.39) & & \text{if}\;0.4\leq \lambda \leq 1.75\\ & +0.139\;\text{sin}(8.04\lambda +2.54)\\ & +0.0018\;\text{sin}(32.2\lambda -6.45)\\ & +0.012\;\text{sin}(23.2\lambda -2.64))\\ 0 & & & \text{otherwise}. \end{array} \end{array}$$ (13)

Here, *σ*_0_ = 2 × 10^5^ Pa is the maximum isometric stress. The active component in the model is also multiplied by a function *a*(*t*) that represents the activation, which increased from 0 to 1 over the course of the contraction. *a*(*t*) is linearly ramped in discrete steps *t*, which we will call “timesteps”. At each step we compute the new state **u**, *p*, and *J* of the muscle. The relationship between the stress and the strain-energy functions is given by

$$\begin{array}{c} \sigma \left(\lambda \right)=\lambda \frac{\partial W(\lambda )}{\partial \lambda }. \end{array}$$ (14)

The strain-energy function for the along-fibre component can then be formulated as

$$\begin{array}{c} W_{\text{fibre}}\left(\lambda \right)=W_{\text{pass}}\left(\lambda \right)+a\left(t\right)W_{\text{act}}\left(\lambda \right). \end{array}$$ (15)

All of the parameters used in the model, and their values are summarized in Table 1. The base material and along-fibre component were implemented in a quasi-static model described in Wakeling et al. [34].

**Table 1:** Summary of parameters used in the model. List of the values for the aforementioned parameters used in this model. *c*_i,cell/ecm_ are the Yeoh model parameters shown in Eq 11 and were obtained using nonlinear regression analysis.

Parameter	Value/Range of Values
*c*_1,cell_	3703
*c*_2,cell_	-707.7
*c*_3,cell_	123.2
*c*_1,ecm_	1988
*c*_2,ecm_	4917
*c*_3,ecm_	-591.5
*α*	2—20%
*s*_ECM_	150—250
*κ*_cell_	1 × 10^6^—1 × 10^8^ Pa
*κ*_ECM_	1 × 10^6^ Pa
*σ*_0_	2 × 10^5^ Pa

## Methods

### Stress-strain experiments

**Fig 2:**
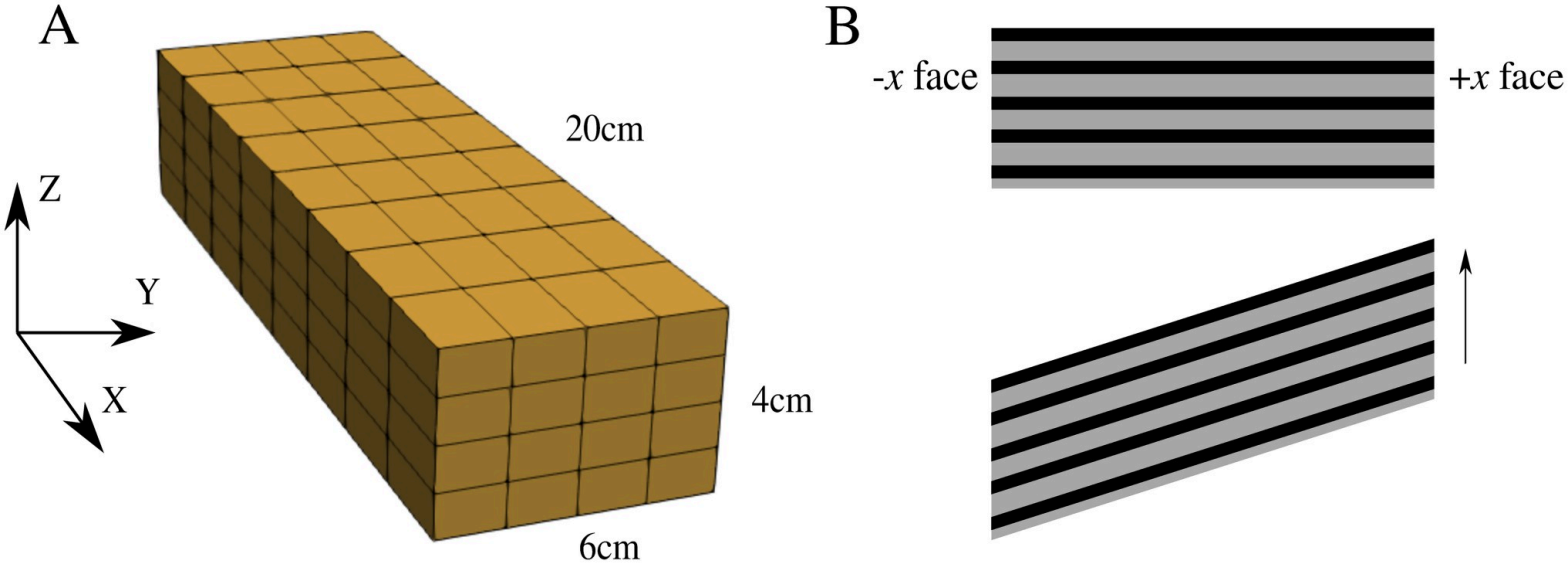
Mesh and experiment setup. (A) Mesh of the geometry used for the numerical experiments. The geometry had dimensions 20cm × 6cm × 4cm and muscle fibre properties are orientated along the *x* axis. (B) Shear experiment setup. The −*x* face was constrained in all directions, while the +*x* face was constrained in the *x* direction only. The arrow represents direction of applied shear stress.

A block of muscle was constructed as seen in Fig 2(A), which had dimensions 20 cm × 6 cm × 4 cm. The fibre properties were implemented along the length of the muscle model in the longitudinal direction (parallel to the *x* axis). To perform stress-strain tests that will allow for a comparison to experimental whole muscle data, the −*x* face was constrained from movement in *x*, *y*, *z* directions. A traction was then applied to the +*x* face of the muscle which extended the muscle in the longitudinal direction.

These tests were performed with varying *α* in the range 0.02—0.20 and *s*_ECM_ coefficients of 150 and 250. The stiffness coefficients were varied to give results that are comparable to experimental data for muscle. The range from 2% to 10% volume fractions of ECM are typically found in healthy muscle [20, 21], and larger volume fractions in the range of 10% to 20% are found in fibrotic tissue [21]. By comparing the stress-strain curves of the model to experimental data, the accurate homogenization parameters, *s*_ECM_ and *α*, can be determined.

### Shear experiments

Shear tests were performed to investigate the behaviour of the model in response to more complex deformation modes. A shear stress was applied to the +*x* face of the model, which was constrained from movement in the *x* direction. To apply the shear stress to the model, we specify the component of the non-zero traction boundary condition in the *y* direction and set the *x* and *z* component of the traction to 0. Meanwhile, the −*x* face was constrained in all directions (Fig 2B). The shear stress was applied in three different scenarios to determine the impact of the base material stiffness and anisotropy in the model: (1) the shear stress was applied without activation with *α* = 0.05 and 0.10, (2) the shear stress was applied prior to activation of the model, and (3) the shear stress was applied after activation of the model.

### Investigation of bulk modulus and strain-energy properties

Muscle is typically considered to be isovolumetric, however, small changes in volume may occur during muscle stretches [46], and also during long fatiguing contractions [32]. Willwacher et al. [32] found that volume changes occured up 9% in the gastrocnemii during running activities, and so to confine volume changes to this range a value for *κ* > 1 × 10^6^ Pa is required. Given the results from previous studies and lack of experimental data for the compressibility of the ECM, the *κ*_ECM_ was left at 1 × 10^6^ Pa [4]. Skeletal muscle consists of 80% water [47], which is contained in the cellular component of the muscle and makes it highly incompressible. Therefore, *κ*_cell_ was varied in the range 1 × 10^6^ to 1 × 10^8^ Pa to look at the effects of the bulk moduli on the stress-strain relationship and strain energy-density distribution. The micro-mechanical components impact the overall stiffness of the base material component, and these effects on the strain-energy distribution were also investigated with *κ*_cell_ = 1 × 10^7^ Pa, *κ*_ECM_ = 1 × 10^6^ Pa, and *s*_ECM_ = 150. To obtain a better understanding of the physics occurring in the model, the volume fractions of ECM were varied between 2% and 100%. The set up for these tests was the same as for the tests for the stress-strain experiments with the addition of an activation phase after the passive lengthening. This involved constraining both the positive and negative *x* faces of the muscle block after the muscle had been passively lengthened, then increasing the activation in the muscle to 100%.

## Results

### Stress-strain results

**Fig 3:**
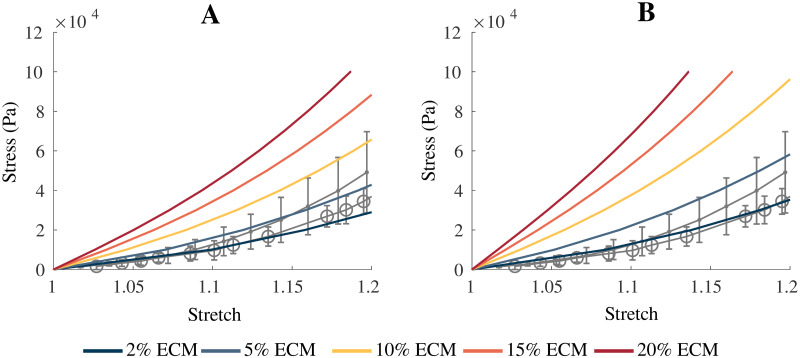
Comparison of model results to experimental data. Comparison of model passive stress-stretch curves to experimental data for skeletal muscle. (A) Gives the model with a parameter of *s*_ECM_ = 150, while (B) is the model with a parameter of *s*_ECM_ = 250. *α* was varied between 0.02—0.20, which corresponds to 2—20% volume fraction of ECM. The grey lines represent experimental data from Takaza et al. [48] (circle) and Mohammadkhah et al. [44] (dot). Error bars represent ± standard deviation when available.

The model qualitatively demonstrates similar stress-stretch behaviour to available experimental data. These data for skeletal muscle vary depending on the species [44, 48], so it is not useful to compare directly to one particular set of muscle data. The stress values from the model are the applied traction to the +*x* face of the block, and the stretch values are the whole muscle stretch

$$\begin{array}{c} \lambda_{\text{muscle}}=\frac{l}{l_{0}}, \end{array}$$ (16)

where *l*_0_ and *l* are the initial length and current length of the muscle belly, respectively. Fig 3 shows that for *s*_ECM_ = 150 and 250 there is a particularly good match at smaller stretch values. Comparable material stiffness to healthy muscle occurs for *α* < 0.10 for *s*_ECM_ = 150 which is a larger range of volume fractions compared to *s*_ECM_ = 250 (< 0.05). However, to better capture the nonlinearity seen at larger strains a larger value of *s*_ECM_ = 250 is required. As the volume fraction of the ECM was increased, there was an increase in stiffness that is expected with fibrotic tissue.

### Shear results

**Fig 4:**
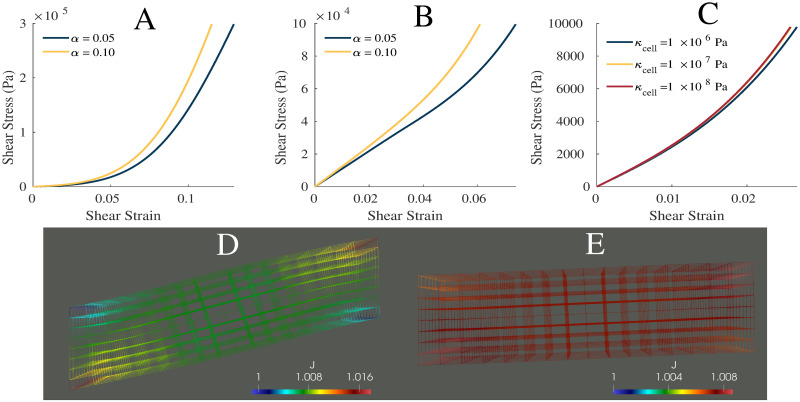
Shear properties. Plot of the shear stress-strain relationship for *α* = 0.05, 0.10 and *s*_ECM_ = 250, while the muscle is passive (A) and active (B). (C) Shear stress-strain relationship for bulk moduli of 1 × 10^6^, 1 × 10^7^, and 1 × 10^8^ Pa. Wire mesh of muscle model after shear stress was applied then model was activated (D), and after first activation then application of shear stress (E). (D,E) Color represents the dilation seen in the muscle model. (C) Shear stress-strain relationship for bulk moduli of 1 × 10^6^, 1 × 10^7^, and 1 × 10^8^ Pa.

The effects of applying a shear stress to the model was demonstrated in Fig 4 with shear strain calcated as

$$\begin{array}{c} \epsilon_{\text{shear}}=\frac{{\bar{u}}_{y}}{l_{0}}, \end{array}$$ (17)

where ${\bar{u}}_{y}$ is the mean displacement in the *y* direction of the +*x* face. At small values of shear strain, there is a linear region in the shear stress-strain relationship and only a small effect from the variation in *α* (Fig 4). This shows there is more influence from the fibres for small shear stresses. At larger strains, the stress response for the model varies with *α*, and the graph becomes more nonlinear, demonstrating the nonlinearity in the base material (Fig 1A). While the model is active, shear stress-strain relationship becomes more linear due to larger fibre forces (Fig 4B). In Fig 4(D) and 4(E), the three dimensional mesh of the model is shown at 100% activation. The largest dilations occur in the corners of the model which experience the most stretching during the shear. Fig 4(D) shows the model during a fixed-length contraction after a shear stress has been applied. The deformation and dilation are smaller, compared to Fig 4(E), where the model has been first activated then sheared.

### Volumetric effects

**Fig 5:**
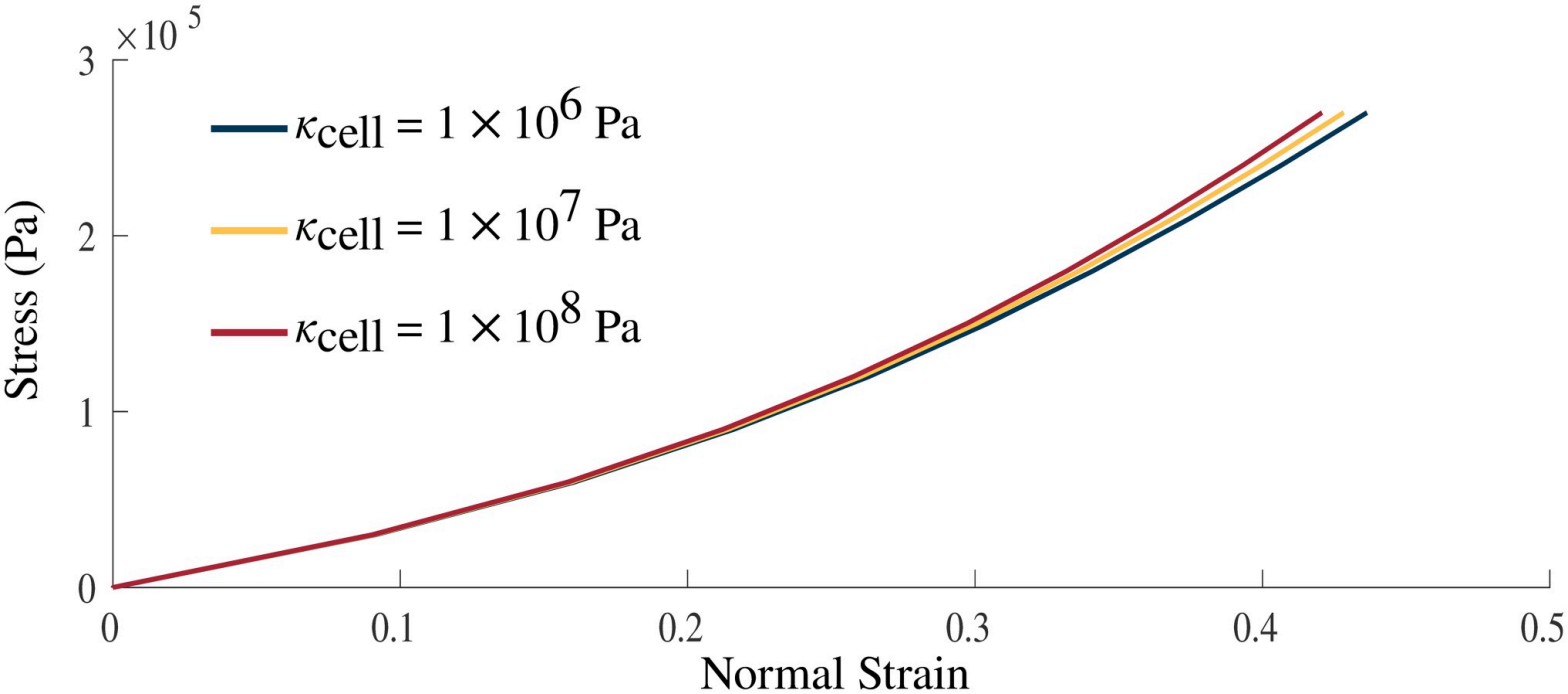
Volumetric impact on stress-strain relationship. Stress-strain relationship with *κ*_cell_ = 1 × 10^6^, 1 × 10^7^, 1 × 10^8^ Pa. Stress was applied in the longitudinal direction on the +*x* face of the muscle model. Increasing values of the bulk moduli result in a stiffer material.

Variation in the bulk modulus of the cellular component shows small effects on the normal stress-strain (Fig 5) and shear stress-strain (Fig 4(C)) relationships. The largest variations in the stress-strain relationships were observed between *κ*_cell_ = 1 × 10^6^ Pa and 1 × 10^7^ Pa whereas smaller variations between the relationships were seen at larger *κ*_cell_. Table 2 gives the volume changes and normalized stresses on the +*x* face of the muscle during the normal stress-strain experiment, where the change in volume was calculated as the ratio between the current volume and initial volume. The volume in its new configuration was calculated as

$$\begin{array}{c} Vol=\int_{V_{0}}\text{det}\left(\text{F}\right)dV_{0}, \end{array}$$ (18)

where *V*_0_ is the initial configuration of the muscle. At larger bulk moduli smaller changes in volume were seen at maximal activation, for *κ*_cell_ = 1 × 10^8^ Pa the change in volume was considerably smaller at 0.1% change in volume compared to the 7.3% change in volume seen for *κ*_cell_ = 1 × 10^6^ Pa. While the changes in volume varied substantially, the effect on the total muscle force was small (Table 2).

**Table 2:** Total volume change and normalized stress on the +*x* face of the muscle after passive lengthening to a stress of 1 × 10^5^ Pa and fixed length contraction to an activation of 100%. The stress was normalized to *σ*_0_. These values are measured with homogenization parameters of *α* = 0.05 and *s*_ECM_ = 250.

*κ*_cell_ (Pa)	Volume Change (%)	Normalized Stress at 100% Activation
1 × 10^6^	7.3	0.875
1 × 10^7^	0.8	0.907
1 × 10^8^	0.1	0.912

**Fig 6:**
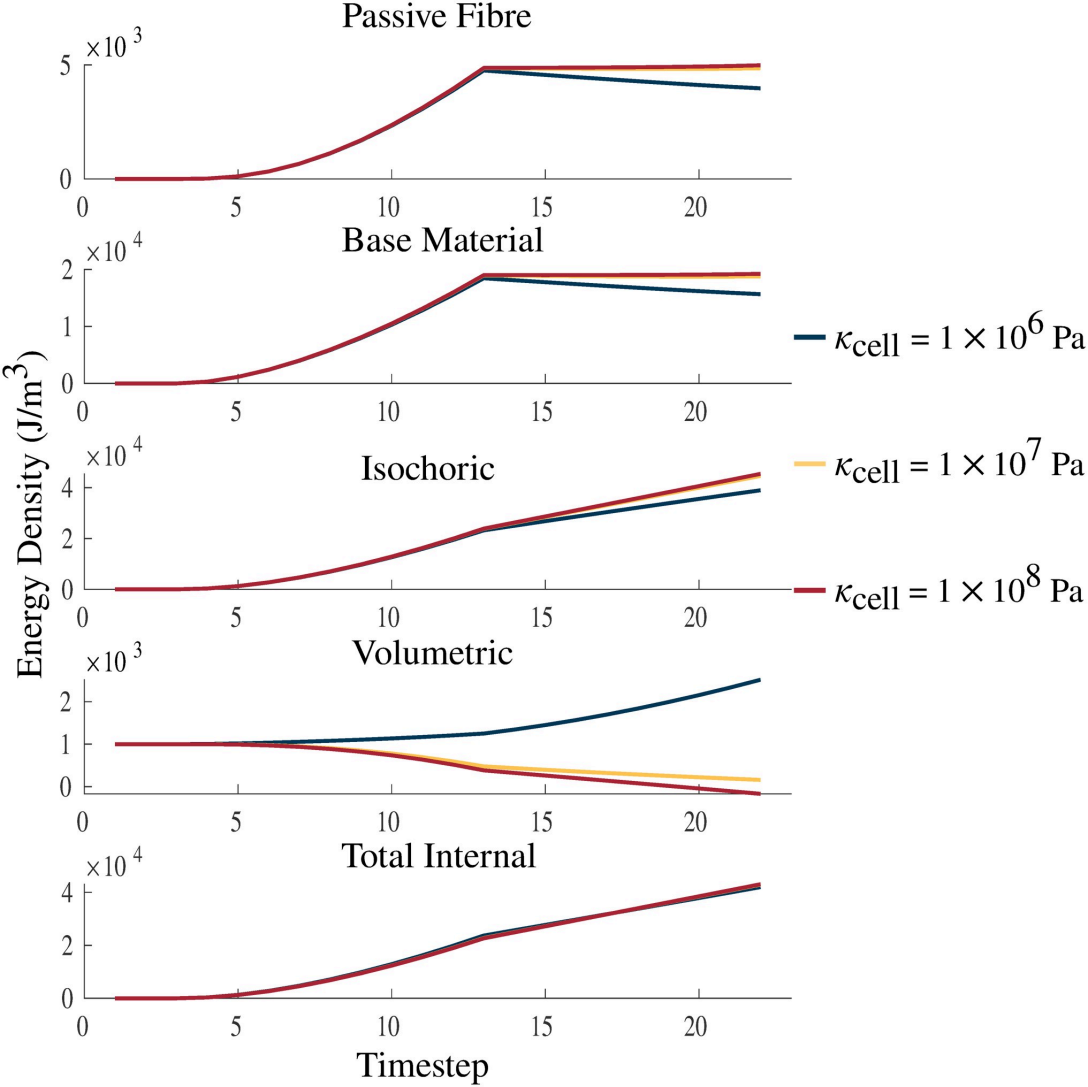
Strain energy-density with varying *κ*_cell_. Plots of passive fibre, base material, isochoric, volumetric, and total internal strain energy-densities. The energies are plotted over a passive lengthening period, up to a traction of 1 × 10^5^ Pa, from timestep 3 to 13, and a linearly increasing fixed-length activation from timestep 13 to 23. *κ*_cell_ is varied between values 1 × 10^6^ Pa, 1 × 10^7^ Pa, and 1 × 10^8^ Pa. The larger values of *κ*_cell_ demonstrate increasing incompressibility and approach the bulk moduli of water (2.15 × 10^9^ Pa [49]), which is considered to be almost completely incompressible. The total internal strain energy-density is the combination of the volumetric, isochoric, and activation (not shown in figure) energies.

Changes in the *κ* of the muscle material also had an impact on the strain energy-density distribution of the model. The strain energy-density calculations were performed as in Wakeling et al. [34]. There was very little change to many of the energy components, in particular, the isochoric components of the energies for the passive lengthening periods of the experiments (Fig 6). There was only a substantial effect to the strain energy-densities in the volumetric component where the energy decreases with increasing bulk moduli. Large effects were seen during activation on the volumetric component with activation increasing the volumetric energy for some values of *κ*_cell_ (1 × 10^6^ Pa) and decreasing the energy for others (*κ*_cell_ = 1 × 10^7^, 1 × 10^8^ Pa). Overall, the total internal energy remains largely unchanged by the value of *κ*_cell_.

### Micro-mechanical impacts on the strain-energy distribution

**Fig 7:**
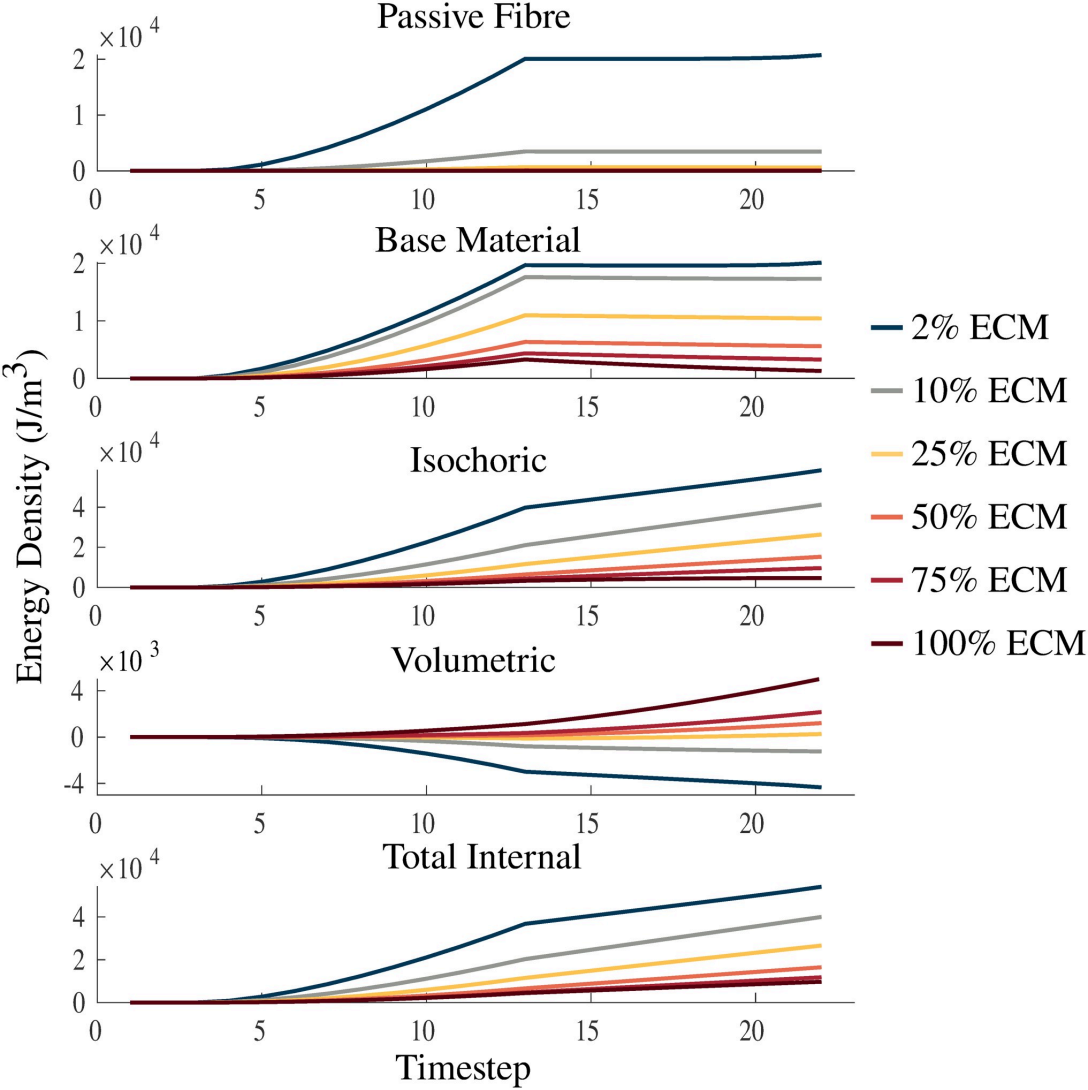
Strain energy-density with varying ECM volume fraction. Plots of passive fibre, base material, isochoric, volumetric, and total internal strain energy-densities. The energies are plotted over a passive lengthening period, up to a traction of 1 × 10^5^ Pa, from timestep 3 to 13, and a linearly increasing fixed-length activation from timestep 13 to 23. Volume fractions of the ECM are varied between 2%, 25%, 50%, 75%, and 100% to investigate the physics of the model.

Fig 7 shows the impact of varying the volume fraction of ECM in the ranges 2-100% on the strain energy-density distribution during a passive lengthening test and fixed-end contraction. As the volume fraction of the ECM becomes larger the muscle becomes stiffer, smaller strains are reached and less deformation occurs, which then results in smaller magnitudes of energy potentials. The volumetric component of the energy decreases as the stiffness in the material decreases, while the opposite behaviour occurs for each of the other components in the material. Additionally, similar effects are seen during activation to the results in Fig 6, where there is increasing volumetric energy for positive volumetric strain-energies and decreasing volumetric energy for negative volumetric strain-energies. In contrast to variations in the bulk moduli (Fig 6), the total internal energy in the model is affected more by variations in the volume fraction of ECM.

## Discussion

### Micro-mechanical properties

The approach taken in this study develops a base material for whole muscle based on the principle micro-mechanical components. This aggregate base material is implemented into a continuum mechanical model developed in previous studies [4, 34]. This homogenized base material showed a good comparison to the experimental data from Takaza et al. [48] and Mohammadkhah et al. [44], with which it was developed. The *s*_ECM_ parameter was varied to account for uncertainty in the experimental data calculation, however, with improved experimental techniques alteration of *s*_ECM_ may not be required. As described previously, the larger values of *s*_ECM_ result in larger nonlinearity in the stress-strain curves for the muscle tissue. This implies the ECM component of the model is largely responsible (along with the anisotropic component) for the nonlinear effects seen in the model, which agrees with experimental data [18, 28]. Binder-Markey et al. [20] found that the ECM volume fractions for various skeletal muscles were typically less than 10%, and for some muscles (eg. Semimembranosus) the volume fractions were less than 2%. Therefore, the volume fractions less than 10% in Fig 3(A) and less than 5% in Fig 3(B) are reasonable ranges when compared to the experimental data for healthy muscle.

While other models have considered an explicit anisotropic ECM [15–17], in the model developed here these effects are accounted for in the one dimensional along-fibre component. Nevertheless, accurate stress-strain mechanics result from the model (Fig 3). A unique component of this model is the use of a nonlinear cellular component derived from brain grey matter. The cellular component of muscle is difficult to measure experimentally, and grey matter provided a good substitute. It demonstrated similar isotropic effects and structure to the cellular component of muscle, and therefore provided good experimental data for the model. Some homogenization methods have considered a linear titin response for the cellular contribution [15, 17], which may not elicit an isotropic response in muscle, or a response derived through a ratio between the fibre and ECM stiffness to ensure they are of the same order of magnitude [15]. Here the model is developed using a different implementation of the cellular component (brain grey matter), and has resulted in realistic behaviour when compared to skeletal muscle (Fig 3). Additionally, when a shear stress was applied to the model, the material is able to capture most typical shear behaviour seen in muscle [15, 50]. At small strains there is a linear relationship and little effect from variations in the base material stiffness. At larger strains, there is more nonlinearity in the shear stress-strain relationship and more effect from the base material properties. This demonstrates the nonlinearity in the base material, and is qualitatively similar to the shear results in other muscle models [15]. The order of magnitude of the shear stress is on the same order of magnitude as that of the normal stress, which agrees with the previous findings [50].

### Volumetric and strain-energy effects

Skeletal muscles are often viewed as nearly incompressible materials [51], however, volume changes that may occur have often been within the error of the measurement device [46] and recent studies have reported volume changes for long fatiguing contractions [32]. Therefore, the volumetric properties of the model were manipulated to determine the effects of varying the bulk moduli in nearly incompressible materials. Fig 5 demonstrates that variations in the bulk moduli have little effect on the overall stress-strain relationship during passive tests, particularly in the physiological range that muscles typically operate over λ_muscle_ < 1.1 [52], which agrees with previous results [53, 54]. This demonstrates that when considering the mechanical behaviour the model there is little dependence on the bulk moduli assuming it is nearly incompressible. [34] suggested that the isochoric and volumetric components of the strain-energy can play a critical role in understanding muscle mechanics on a three dimensional level. When considering the distribution of the strain energy-densities there is an effect from the bulk moduli of the material. The total potential energy in the system, including the energy from activation and external work on the material, is balanced during the quasi-static simulation ran in this study. As the material became more incompressible, the volumetric strain energy-density decreases counteracting the increase in energy seen in the isochoric component of the total strain-energy. The increases in isochoric strain-energy occured due to increased strain during the passive lengthening phase. These impacts on the energies are likely due to a greater energetic penalty associated with volume change. The isochoric components of the strain energy-density distribution (passive fibre and base material) appear nearly unaffected, which can be explained by the difference in magnitudes of the strain-energy. However, the distribution of the strain energy-densities was impacted by the choice of bulk moduli, which contributed to the energy balance in muscle and the ability to resist volume change. The total contractile force produced by the muscle during the fixed-end contraction was not strongly impacted by the bulk moduli (Table 2). The main effect from increasing the bulk moduli was in the decreased ability of the material to change volume.

Investigation of the strain-energy distribution in the muscle model allows for an understanding of muscle behaviour during deformation and contraction. Fig 7 shows that as the muscle is pulled to a traction of 1 × 10^5^ Pa there is a strong energy dependence on the stiffness of the material. The results show that there is larger internal energy developed by the material with lower stiffness (2% ECM), which is expected given that compliant tissue will have a larger strain. Interestingly, there are negative volumetric strain energy-densities that appear during passive lengthening and activation. This is due to the calculation of the strain energy-densities, which are calculated with respect to the initial configuration in which the energy is assumed to be zero for all the components. Therefore, negative values are expected for the balance of the energies. The increasing activation in the muscle had a relatively small impact on the passive fibre and base material energies (Fig 7), likely due to the constraints imposed during fixed-length activation restricting movement of the ±*x* faces of the geometries. Large variations occur in the volumetric and isochoric energies in Fig 7, and are partly due to the difference in bulk moduli of the micro-mechanical components (similarly to Fig 6). Although, the stiffness of the material does play a significant role in increasing the variation in energy for varying volume fractions. By investigating the effects of the strain energy-density distributions, an understanding of how the stiffness of the material, which can be altered through the homogenized model, impacts the energy lost or gained through a three dimensional muscle architecture. In this case the increase in stiffness of the material was shown to increase the volumetric energies and hence reduce the ability of a muscle to deform or bulge during contraction. This in turn gives an understanding of how the combination of the microscopic composition and macroscopic deformation of the muscle impact the distribution of strain energy-densities, which demonstrates the critical role these material properties play in contributing to the force produced by skeletal muscle. The micro-mechanical parameters demonstrated a strong impact on muscle mechanics, while *κ*_cell_ had a strong effect on the model’s ability to change volume, the volume fraction of the ECM, *α*, was particularly important in altering the strain energy-density distribution.

### Applications

Homogenization models are used to analyze the impacts of the variation of micro-mechanical components, and have the ability to investigate the effects from many conditions such as fibrosis. Fibrosis is the increase in collagen content in the muscle as the result of diseases such as cerebral palsy or muscular dystrophies [22, 28]. This model allows for the investigation of these effects by altering the volume fraction of the ECM. Fig 7 shows that alterations in the volume fraction of the material have strong effects on the mechanics of the muscle, and further investigation of varying micro-mechanical properties and the impacts on the strain energy-density distribution could provide a deeper understanding of the effects from fibrosis. The stiffness parameter for ECM, *s*_ECM_, allows for investigation of effects such as glycination, which is a type of biochemical linkage between a sugar and a protein or lipid. In the process of aging, glycination occurs which increases the stiffness of the ECM [55], and these effects could be further understood through an application of this model. The formulation of forces and energies in the model allows for an analysis of the contribution from each of the homogenized components to the whole muscle mechanics. This ability to analyze the impacts from individual components is not typically found in macroscopic models, but can provide insight into the mechanics of muscles under conditions of varying micro-mechanical properties.

## Conclusion

In this paper we have developed a principled model for muscle base material, which has been designed to easily encorporate available micro-mechanical experimental data from the literature into a macroscopic model. The characteristics of this model were then examined through tension and activation experiments for both normal stress and shear stress experiments. The breakdown of the strain energy-densities associated with passive lengthening and activation were analyzed under the effects of micro-mechanical components, and these components were found to have an effect on the distribution of these energies. This numerical model has the potential for gaining a deeper understanding on the effects of changes to the tissue micro-structure and composition on the three dimensional mechanics of muscle contraction.

## Supporting information

S1 Appendix Finite element method description. (PDF)
