# Supplementary material for: Modelling extracellular matrix and cellular contributions to whole muscle mechanics
Source: PLoS One. 2021 Apr 2;16(4):e0249601. doi: 10.1371/journal.pone.0249601 (PMC8018661; doi:10.1371/journal.pone.0249601)
Supplement: S1 Appendix — (PDF) [file pone.0249601.s001.pdf]

S1 Appendix

Finite Element Method Description

A block geometry of size 20cm×6cm×4cm was used in this study. Zero displacement boundary conditions were applied to the $-x$ face of the block in the x, y, z directions. A non-zero traction boundary condition was applied to the $+x$ face of the block, while zero traction boundary conditions were applied everywhere else.

The finite elements spaces for the unknowns are $(\mathbf{u}, p, J) \in \mathbf{Q}_{k+1} \times P_k \times P_k$. Where $\mathbf{Q}_{k+1}$ and P_k are defined as

$$\mathbf{Q}_{k+1} := \left\{ \mathbf{v} \in \mathbf{C}(\overline{V}_0) : \mathbf{v}|_T \in \mathbf{P}_{k+1}(T), \ \forall T \in \mathcal{T}_h \right\} \cup \mathbf{H}^1_{\Gamma_0}(V_0), \tag{1}$$

$$P_k := \left\{ q \in L^2(V_0) : q|_T \in P_k(T), \ \forall T \in \mathcal{T}_h \right\}. \tag{2}$$

$\mathcal{T}_h$ is a regular triangulation of $\overline{V}_0 = V_0 \cup \partial V_0$ by a hexahedral T with a diameter of h_T, where V_0 is the initial configuration and ∂V_0 is the boundary of the inital configuration. We let the mesh size h be defined as $h := \max\{h_T : T \in \mathcal{T}_h\}$. $\mathbf{P}_k(T)$ is the vector version of $P_k(T)$. $\mathbf{C}(\overline{V}_0)$ denotes the set of continuous function on $\overline{V}_0$. $\mathbf{H}^1_{\Gamma_0}$ is defined as

$$\mathbf{H}^1_{\Gamma_0} := \{\mathbf{v} : \mathbf{v} \in L^2(V_0), \nabla \mathbf{v} \in L^2(V_0), \mathbf{T}\mathbf{v} = 0 \text{ on } \Gamma_0\}, \tag{3}$$

where $\mathbf{T}$ denotes the Dirichlet trace operator and $\nabla \mathbf{v}$ denotes the gradient of $\mathbf{v}$. Γ_0 is the region of the boundary with zero Dirichlet conditions, and in this study, this corresponds to the $-x$ face of the muscle block. The space $L^2(V_0)$ is the defined as

$$L^2(V_0) := \{\mathbf{v} : \int_{V_0} |\mathbf{v}|^2 dV < \infty\}. \tag{4}$$

In this study, we set $k = 1$ and integrals in the method were computed using Gaussian quadrature of order 3.

The nonlinear problem was linearized using a Newton-Raphson method. The linear problem was then solved using a Conjugate Gradient Method and a SSOR preconditioner with a relaxation constant of 0.65.

More details on the mathematical model and the numerical implementation, as well as the convergence of the method are given in [1]. Details on the mathematical model can also be found in [2, 3]. The finite element method used in this study was based on [4].

References

[1] Domínguez S. From eigenbeauty to large-deformation horror. Ph.D. Thesis, Simon Fraser University. 2020. Available from: http://summit.sfu.ca/item/20968

[2] Rahemi H, Nigam N, Wakeling JM. Regionalizing muscle activity causes changes to the magnitude and direction of the force from whole muscles-a modeling study. Front Physiol. 2014;5 AUG(August):1–10. doi:10.3389/fphys.2014.00298.

[3] Wakeling JM, Ross SA, Ryan DS, Bolsterlee B, Konno R, Domínguez S, et al. The Energy of Muscle Contraction. I. Tissue Force and Deformation During Fixed-End Contractions. Front Physiol. 2020;11. doi:10.3389/fphys.2020.00813.

[4] Pelteret JP and McBride A. The deal.II tutorial step-44: Three-field formulation for non-linear solid mechanics. Zenodo. 2012. 10.5281/zenodo.439772
